# Supplementary material for: Inhibition of HPSE/SDC-2 axis-induced epithelial-mesenchymal transition for treating IC/BPS
Source: PLoS One. 2025 May 23;20(5):e0321730. doi: 10.1371/journal.pone.0321730 (PMC12101628; doi:10.1371/journal.pone.0321730)

Figure 1B

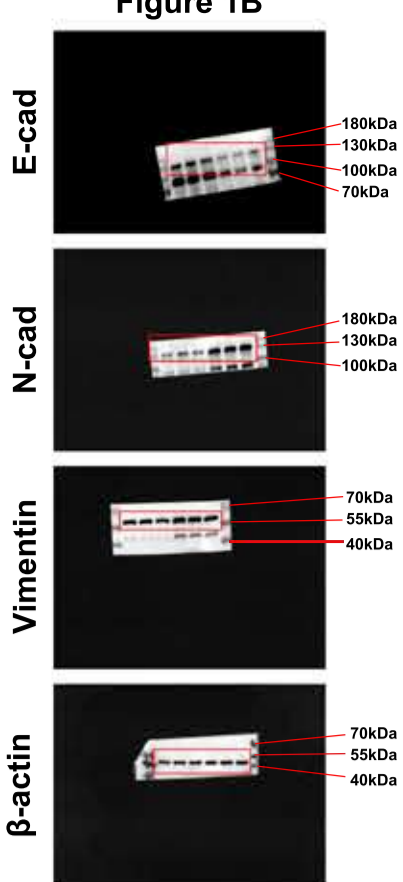

Figure 1E

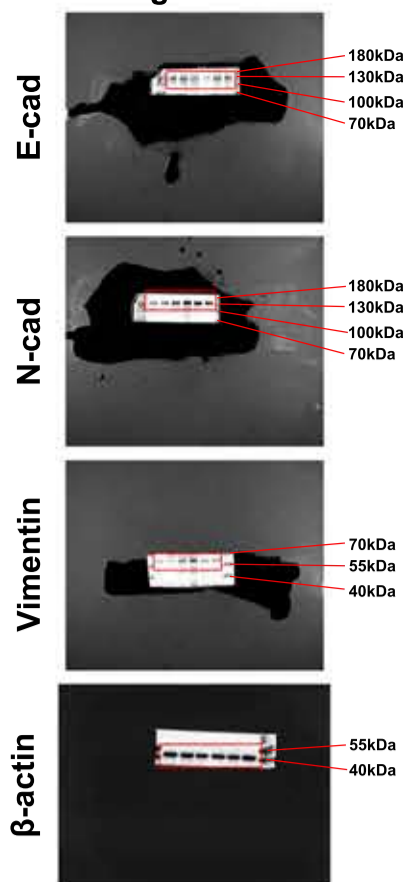

Figure 1H

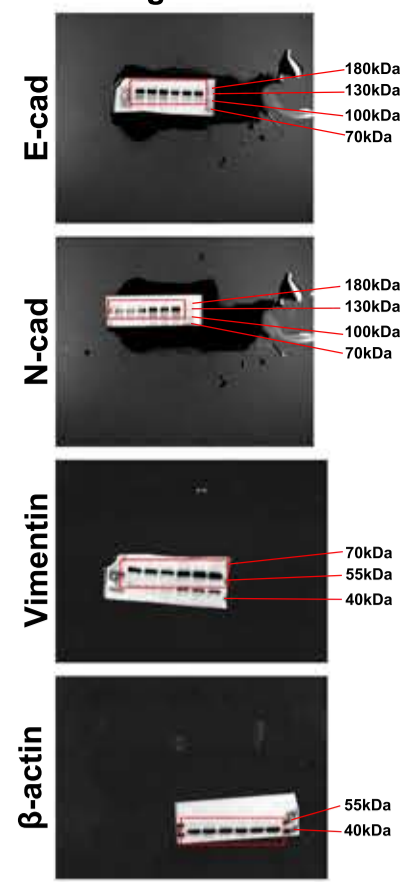

Figure 2C

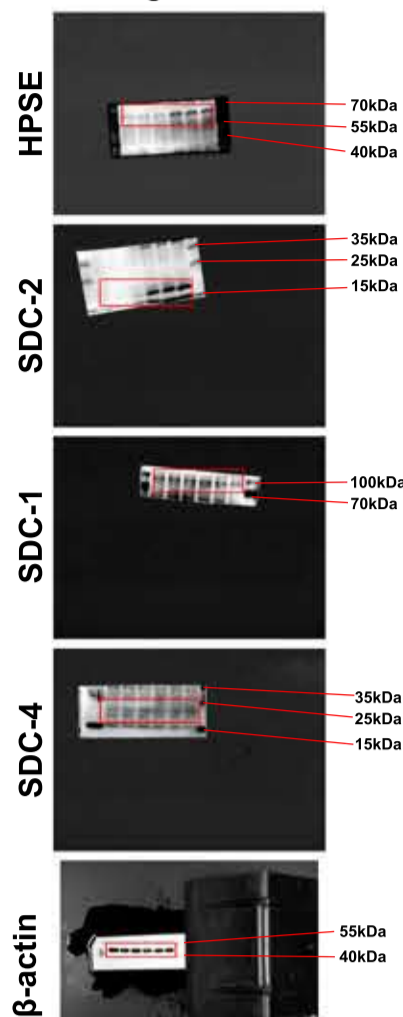

Figure 3A

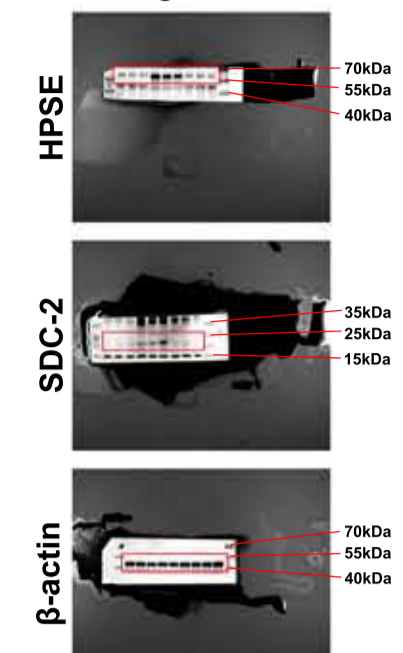

Figure 3E

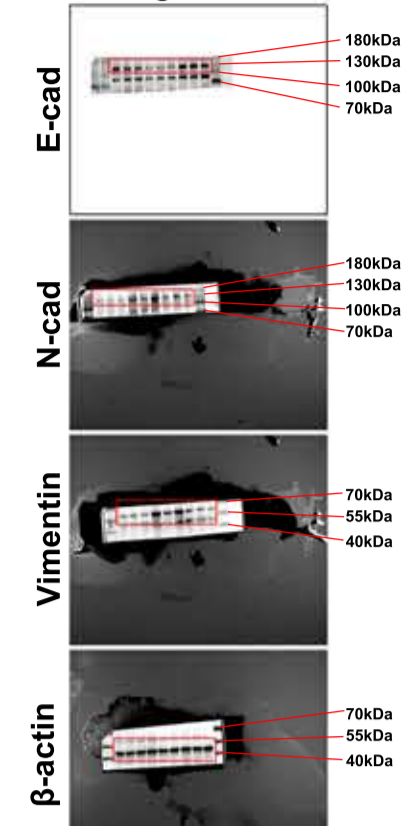

Figure 4A

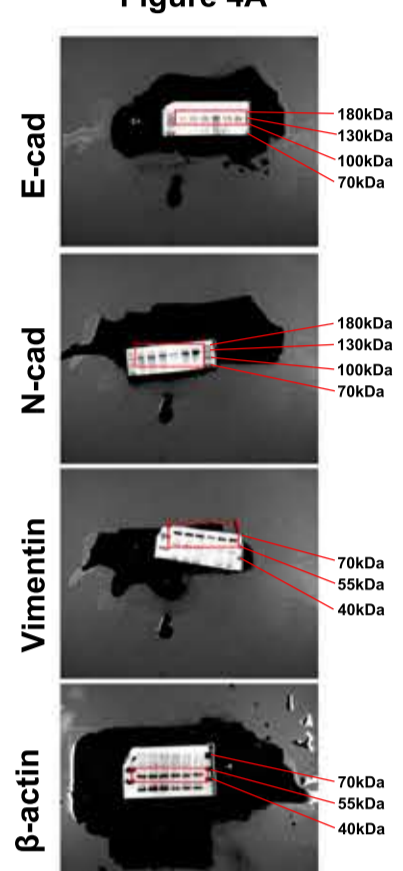

Figure 40

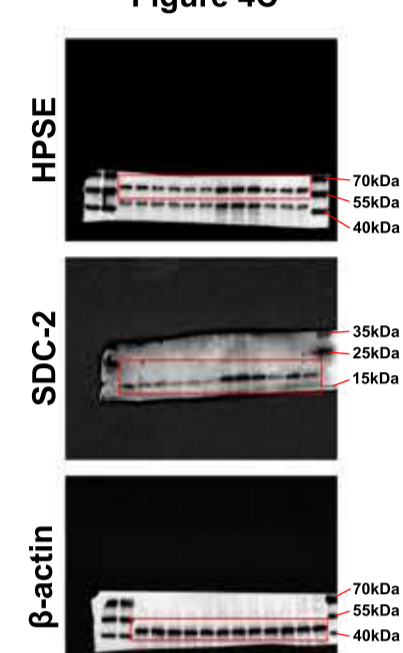

Figure 4D

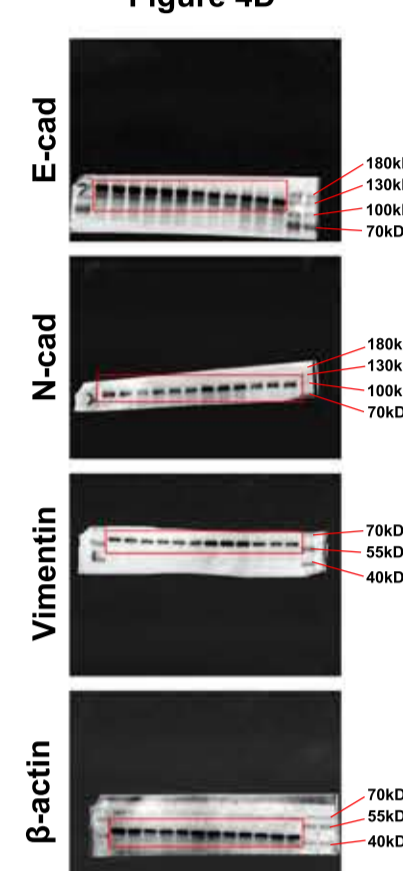

Figure 5B

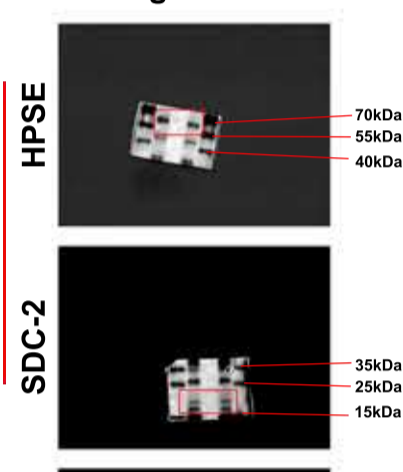

Figure 5D

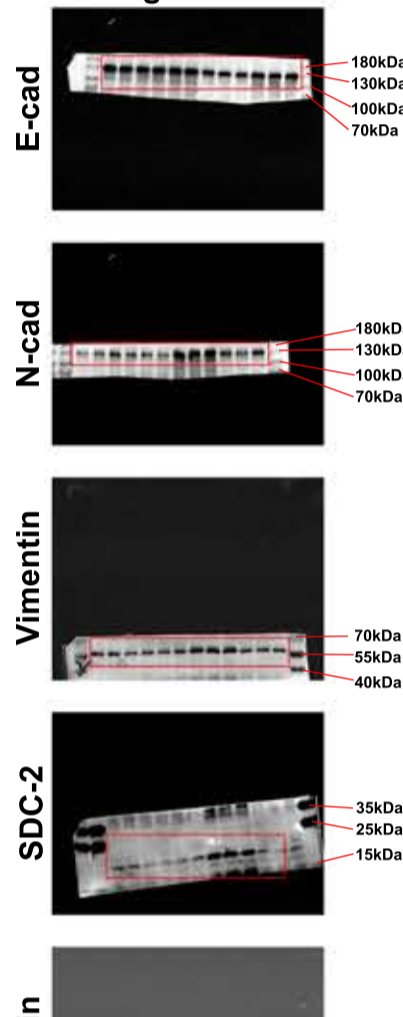

Figure 6

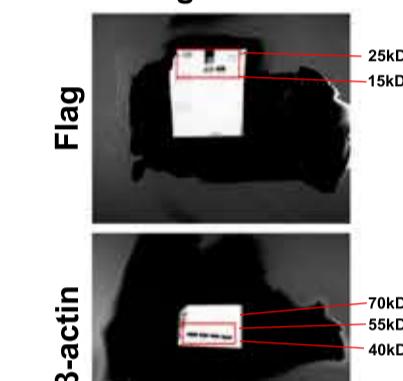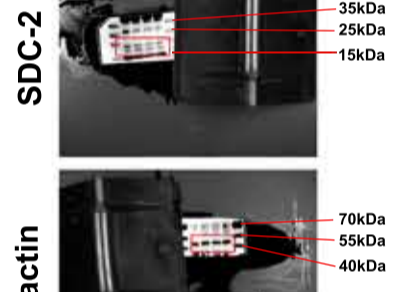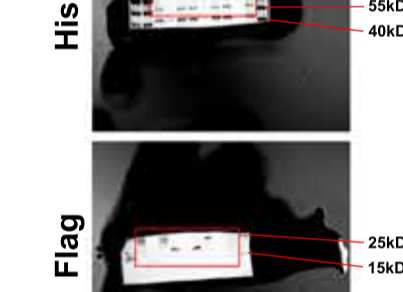

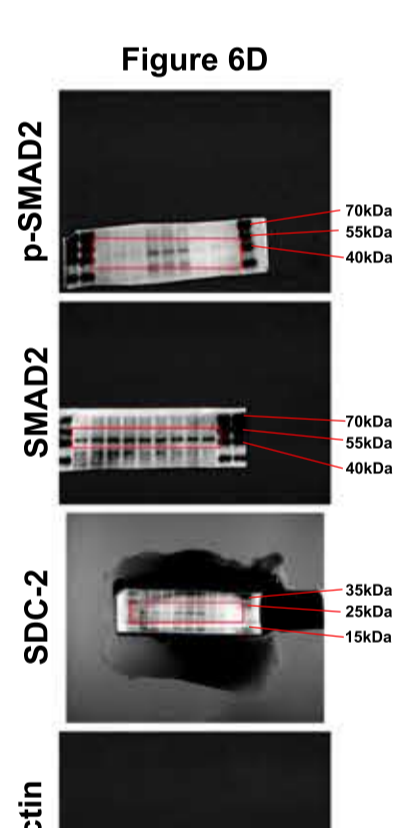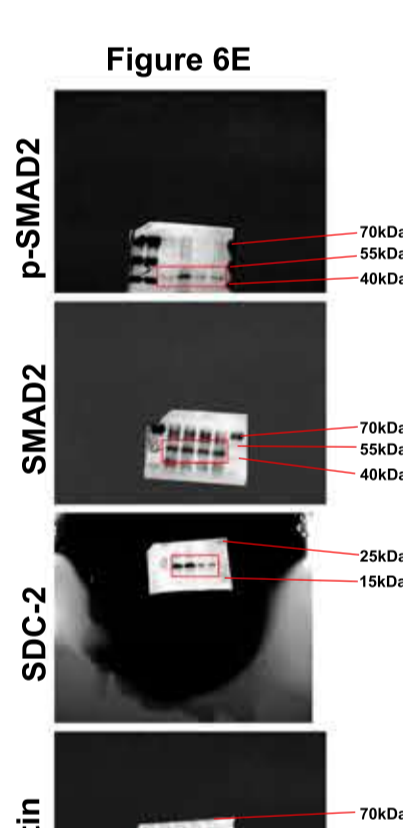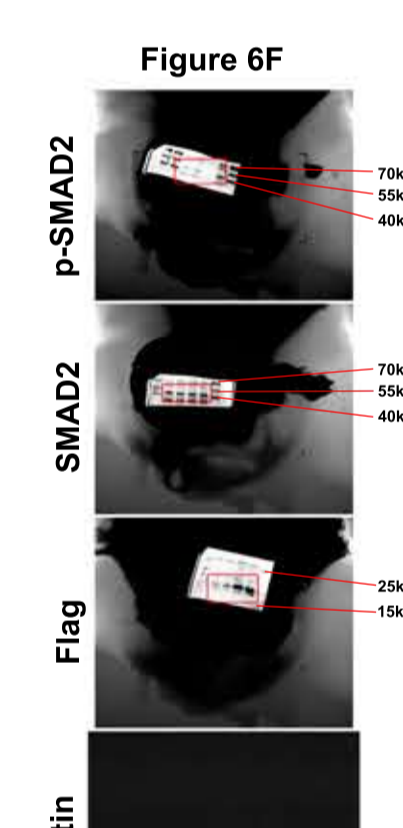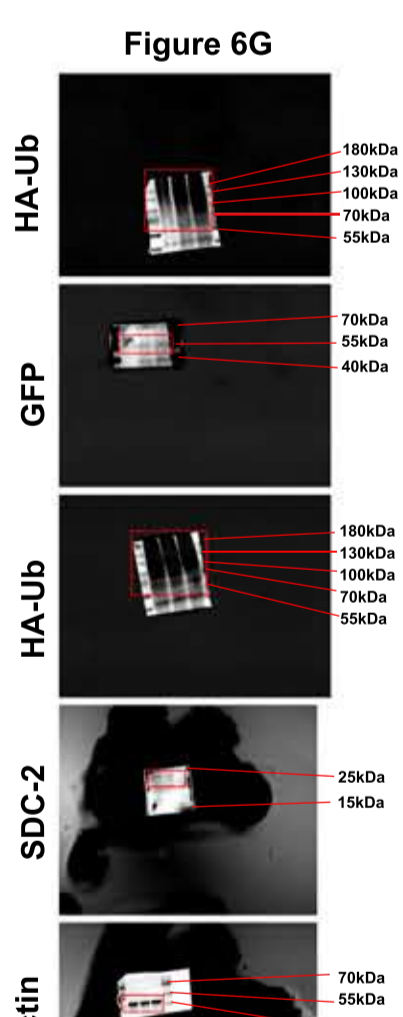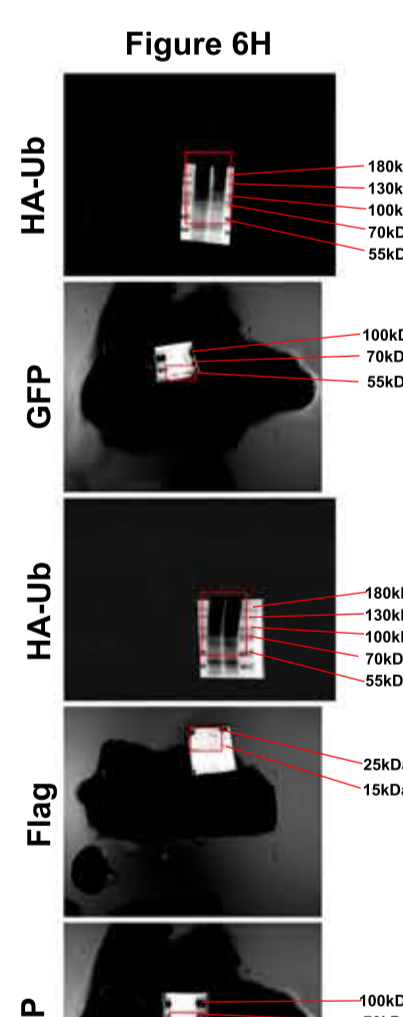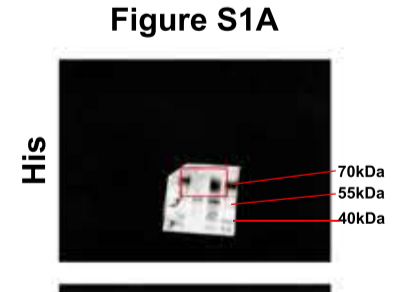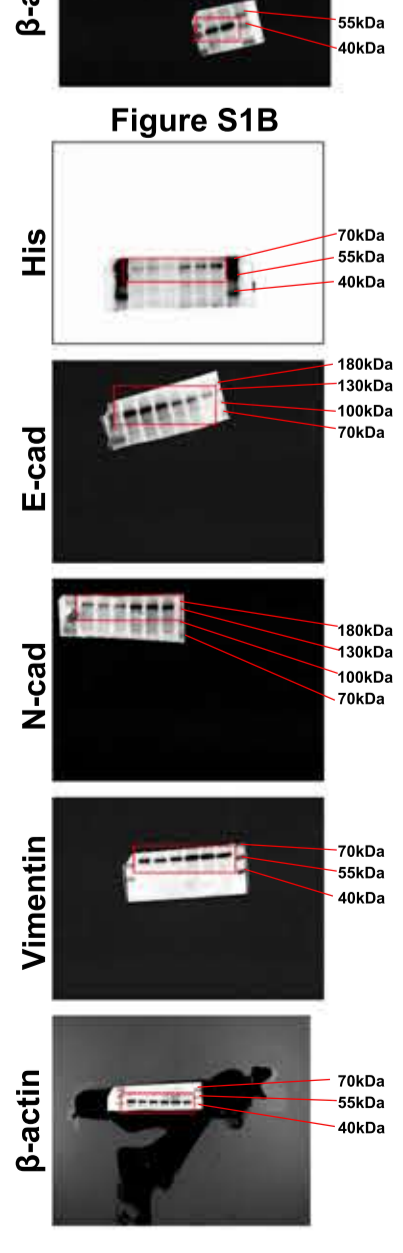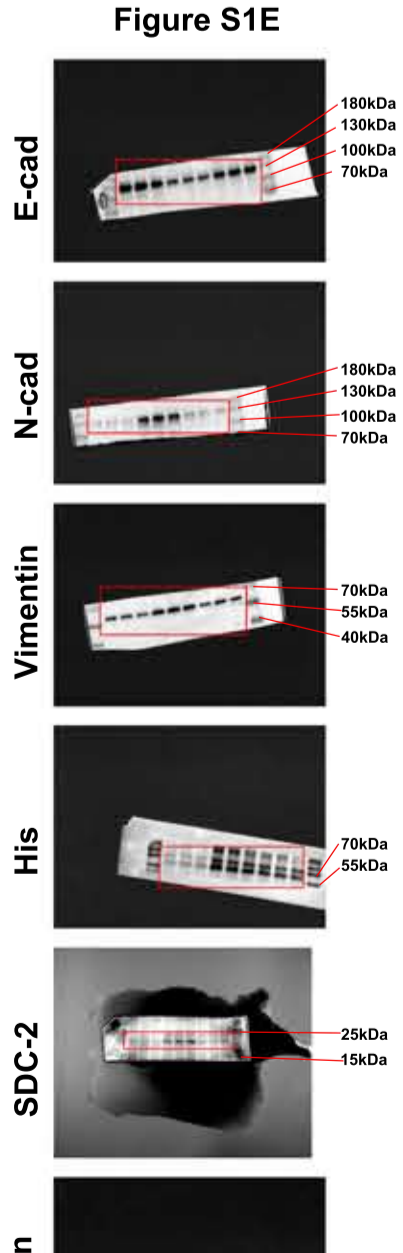

Supplement: S1 Fig — A: WB validation of transfection of His-HPSE. B: WB results of EMT metrics after transfection of His-HPSE. C: IF results of EMT metrics after transfection of His-HPSE. D: Relative fluorescence intensities of leakage occurring after transfection of His-HPSE (n=6). E: WB results of EMT metrics after transfection of WB results of EMT metrics after transfection of His-HPSE and SDC-2-siRNA. F: Immunofluorescence results of EMT metrics after transfection of His-HPSE and SDC-2-siRNA. G: Relative fluorescence intensity of leakage occurring after transfection of His-HPSE and SDC-2-siRNA (n=6). Statistical analyses between two groups were performed by t-test and between three groups by one-way ANOVA, with error line as mean ±standard error, ****P < 0.0001. NC: Negative control. (XLX) [file pone.0321730.s001.pdf]
