# Supplementary material for: Inhibition of HPSE/SDC-2 axis-induced epithelial-mesenchymal transition for treating IC/BPS
Source: PLoS One. 2025 May 23;20(5):e0321730. doi: 10.1371/journal.pone.0321730 (PMC12101628; doi:10.1371/journal.pone.0321730)
Supplement: Attachment — Submitted filename: Response to Reviewers.docx [file pone.0321730.s003.docx]

Dear Editors and Reviewers:

Thank you for your letter and for the reviewers’ comments concerning our manuscript entitled “Inhibition of HPSE/SDC-2 axis-induced epithelial-mesenchymal transition for treating IC/BPS” (ID: PONE-D-24-45975). Those comments are all valuable and helpful for revising and improving our paper, as well as the important guiding significance to our research. We have studied the comments carefully and have made corrections, which we hope to meet with approval.

The revisions in the manuscript are in red fonts, and responses to the reviewers' comments are in blue fonts.

Reviewer #1: In their study, Chen et al. describe the involvement of the HPSE/SDC-2 axis in the development of epithelial-to-mesenchymal transition (EMT) in interstitial cystitis/bladder pain syndrome (IC/BPS) and propose a new therapeutic target for this pathology. In vivo and in vitro experiments were conducted to test the small molecule OGT2115, which was confirmed to inhibit the EMT process. The in vitro studies indicate the potential involvement of HPSE and SDC-2 in the development of EMT and suggest that their inhibition may facilitate the restoration of a favorable phenotype. The authors conclude that OGT2215, an HPSE inhibitor, may represent a promising therapeutic molecule for IC/PBS.
I have a few comments/suggestions to improve and strengthen the study:
• The authors used OGT2115, a HPSE-1 inhibitor, to unveil HPSE-1 role on EMT. The choice of 5 µM as an optimal drug concentration was based solely on EMT phenotypic changes (decrease of E-cadherin and increase of Vimentin at protein level). However, Western blots of Fig 4A-B do not show a strong inhibition of HPSE-1 expression at this concentration. What is the IC50 of OGT2115 on HPSE-1 expression?
Response to comment: Thanks for the comments. OGT2115 (HY-100898, MCE) is a reliable HPSE-1 inhibitor, and its product description indicates that it has an IC50 of 0.4 μM for HPSE-1 inhibition and an IC50 of 1μM for antiangiogenicity. Our reading of the literature has revealed that most of the intervening concentrations of OGT2115 used at in vitro levels are 1 μM ( PMID: 32608014, PMID: 31327483). However, in our pre-experiment results, we found that OGT2115 did not significantly improve the EMT occurring in Sv-huc-1 when intervened at 1 μM, which may be due to the difference in cell lines, different cell culture conditions, etc. Therefore, we set up 0,1.25, 2.5, 5, 10 and 20μM concentration gradients to explore the optimal action concentration of OGT2115 for EMT improvement. According to the purpose of our study, i.e., the ameliorating effect of OGT2115 on EMT, we chose an intervention concentration of 5μM and verified the inhibitory effect of OGT2115 on the HPSE/SDC-2 axis at this concentration in Figure 4C. The reviewer's suggestion was very inspiring, and we will plot IC50 curves directly against the drug's target of action in future studies.

• It is important to perform cytotoxicity assays of OGT2115 to confirm the non-cytotoxic effect of the drug.
Response to comment: Thanks for the comments. We determined the cytotoxicity of OGT2115 using the CCK-8 assay. The results showed that for Sv-huc-1, the IC50 of OGT2115 was 36.26 μM. 5 μM is a safe intervention concentration that does not significantly inhibit cell viability.

• Title and conclusion of the 3.4 paragraph are not very clear. Is there an improvement or an inhibition of the EMT process under OGT2215 treatment? Clarify your message.
Response to comment: Thanks for the comments, and we have described this result more clearly.

• The authors focused on the HPSE/SDC-2 axis implication by modulating HPSE (through OGT2115) but to strengthen and confirm their observations, the use of SDC-2 inhibitors could be interesting. Similarly, the authors performed RNAi experiments with only SDC-2 RNAi. It would be interesting to genetically modulate HPSE expression (by siRNA), deplete both SDC-2/HPSE and finally rescue their expression, to confirm that both proteins are essential.
Response to comment: Thanks for the comments. We searched for SDC-2 inhibitors in the preliminary stage but did not find any product in sale. We refined the overexpression of HPSE and the related response experiments according to the reviewers' suggestions. The experimental results are elaborated as follows:

“We overexpressed HPSE and validated it using WB (Figure S1A). Overexpression of HPSE promoted EMT progression, epithelial dysfunction, and increased fluorescence leakage occurred (Figure S1B-D). We knocked down SDC-2 based on overexpression of HPSE (Figure S1E). The EMT-promoting effect exerted by overexpression of HPSE was counteracted by SDC-2 knockdown (Figure S1E-F). The epithelial function was restored and fluorescence leakage was reduced after the SDC-2 knockdown (Figure S1G). Combined with the previous results, it can be concluded that HPSE binding to SDC-2 caused downstream EMT development and epithelial dysfunction, with SDC-2 playing a key role.”

• In their study, the authors analyzed GSE57560 dataset and described an upregulation for SDC-2 but also for SDC-1 and -4. However, they did not go further with these receptors because there are not significant changes in mice. Can the authors discuss more on that aspect? What is the difference between human and mice for SDC expression?
Response to comment: Thanks for the comments. Mice are able to mimic human pathophysio -logical processes to some extent, but the distribution and function of some proteins such as the SDC family in the bladder are not identical. We analyzed human bladder tissue high-throughput sequencing data and found that the HPSE/SDC axis may play a role in the disease process. From the experimental results, human bladder tissues SDC1, SDC4 were also significantly elevated and had higher expression during the disease process of IC. We found low and no significant difference in SDC1, and SDC4 expression in the mouse model.

We observed the correlation between EMT and urination in mice from a mouse model. The role of EMT on phenotype was verified from the perspective of altered urinary habits. This part of the experimental results of altered urination habits provided by the mouse model is not available in human cell lines, so we will choose SDC-2, which is expressed differently and significantly in mice, for subsequent studies. We look forward to observing the role of the HPSE/SDC-2 axis in the altered urinary behavior of mice in inflammatory states by treating the mice again accordingly, provided that the in vitro levels are verified exactly.
• As they use human cell lines, it could be interesting to perform some experiments in vitro on SDC-1 and -4. Do they interact with HPSE? Are they involved in EMT process? Can they partially replace SDC-2 when SDC-2 is knockdown?
Response to comment: Thanks for the comments. As stated above we chose SDC-2 for subsequent experiments, and EMT was significantly suppressed and restored to near-normal levels when we treated SDC-2 with knockdown (Figure 5D-F). This result suggests that SDC-2 is a key intermediate protein for HPSE to play a role in promoting EMT and is consistent with the results of the mouse model. We strongly agree with the reviewer's suggestion to refine the alternative roles of other proteins of the SDC family in the SDC-2 knockdown state, and we will further refine this part of the relevant content in subsequent studies.
• Finally, they only used phenotypic markers to describe the EMT process (E-cadherin as epithelial marker and Vimentin for mesenchymal marker), but they did not control cytokine expression, already described in the literature to be involved in this process (e.g. TGF-β). In the discussion part, the authors suggest that HPSE can activate TGF-β (without citation). Regarding these informations, it could be interesting to add a TGF-β inhibitor to confirm the EMT induction is TFG-β -dependent.

Response to comment: Thanks for the comments. We subsequently refined the downstream mechanistic studies that SDC-2 is able to activate the TGF-β pathway by binding to SMAD7 and reducing TGFβR1 ubiquitination degradation. This part has been added to the manuscript:

“The TGF-β pathway is an important pathway for promoting EMT and a downstream signaling pathway for HPSE. We found that it was significantly activated in the LPS intervention group and inhibited after the knockdown of SDC-2 (Figure 6D). Then we exogenously added TGF-β1 as a pathway activator, and we found that the activation of the pathway by TGF-β1 disappeared after SDC-2 knockdown (Figure 6E). The activating effect of TGF-β1 was further enhanced after overexpression of SDC-2 (Figure 6F). This result suggests that the activating effect of SDC-2 on the TGF-β pathway is not through the promotion of TGF-β1 secretion, but may exert its biological function through its receptor. Ubiquitination is one of the pathways that promote protein degradation, and we examined the level of ubiquitination of TGF-βR1 under knockdown and overexpression of SDC-2. The results showed that the ubiquitination level of TGF-βR1 increased after the knockdown of SDC-2, and the ubiquitination level of TGF-βR1 decreased after the overexpression of SDC-2 (Figure 6G-H). Taken together, we can conclude that SDC-2 can inhibit TGF-β1 ubiquitination degradation thereby activating the TGF-β pathway.”

Reviewer #2: The manuscript investigates the role of HPSE (heparanase) and SDC-2 in epithelial-mesenchymal transition (EMT) within the context of interstitial cystitis/bladder pain syndrome (IC/BPS), proposing that the inhibition of the HPSE/SDC-2 axis could mitigate EMT-related epithelial dysfunction. While the study presents intriguing data and utilizes multiple methodologies (in vivo, in vitro), there are critical areas where improvements are necessary :
in the introduction section :
• It might be helpful to add specific epidemiological data on IC/BPS, like prevalence or challenges in diagnosis. Including a bit more detail on current treatments and their limitations could help to show why targeting EMT and HPSE/SDC-2 might offer something new in terms of therapy.

Response to comment: Thanks for the comments, and we have added this section.
• Adding a few lines on the functions of HPSE and SDC-2, especially their roles in extracellular matrix remodeling and EMT, might help readers unfamiliar with these molecules to understand why they matter in IC/BPS

Response to comment: Thanks for the comments, and we have added this section.
• The hypothesis is implicit rather than clearly stated. While the introduction discusses the potential involvement of HPSE and SDC-2 in EMT in IC/BPS, it lacks an explicit hypothesis that clearly directs the study's objectives. e.g., “We hypothesize that the HPSE/SDC-2 axis promotes EMT in IC/BPS and that its inhibition can ameliorate EMT-associated epithelial dysfunction.”.

Response to comment: Thanks for the comments. We have formulated hypotheses in the original article to explicitly guide the research objectives.
• You should add a few more references specifically for EMT in bladder disease could strengthen your argument. For exemple, citing related literature on EMT in non-cancerous inflammatory conditions would more enhance the study’s novelty.

Response to comment: Thanks for the comments. We have added EMT-related references.
Methodology and results
• The Results section should be restructured for clarity and logical flow. Each sub-section should begin with a brief statement of specific objectives, followed by a concise summary of the methods used.

Response to comment: Thanks for the comments, and we have made amendments.
• Also, there is sometimes disorganized presentation of findings, for exemple, the abrupt presentation of EMT in bladder tissues, before first confirming cystitis validation, disrupts the logical progression of the results. This sequence should ideally be reversed to establish the model before discussing EMT induction.
Response to comment: Thanks for the comments. We placed the phenotypic validation at the front of the narrative to refine the logic of the study.
• Although the manuscript discusses the HPSE/SDC-2 axis's role in EMT, the mechanistic insights are somewhat superficial. The focus is heavily on EMT marker changes without adequately addressing the epithelial cells’ functional outcome, specifically related to cystitis. More extensive exploration on downstream signaling pathways related to HPSE and SDC-2 in EMT, perhaps involving other known mediators, could strengthen the paper’s mechanistic foundation and relevance for IC/BPS treatment.

Response to comment: Thanks for the comments.We are very sorry that we may have been misleading in the labeling of some experimental sections. Our functional verification of the epithelial cells was mainly done by the epithelial permeability assay, which is the measurement of their anti-leakage function to fluorescent reagents 24h after cell passaging. High fluorescence intensity represents that more fluorescence leakage occurs and epithelial dysfunction.

We explored the downstream pathways and describe them below in the manuscript:

“The TGF-β pathway is an important pathway for promoting EMT and a downstream signaling pathway for HPSE. We found that it was significantly activated in the LPS intervention group and inhibited after the knockdown of SDC-2 (Figure 6D). Then we exogenously added TGF-β1 as a pathway activator, and we found that the activation of the pathway by TGF-β1 disappeared after SDC-2 knockdown (Figure 6E). The activating effect of TGF-β1 was further enhanced after overexpression of SDC-2 (Figure 6F). This result suggests that the activating effect of SDC-2 on the TGF-β pathway is not through the promotion of TGF-β1 secretion, but may exert its biological function through its receptor. Ubiquitination is one of the pathways that promote protein degradation, and we examined the level of ubiquitination of TGF-βR1 under knockdown and overexpression of SDC-2. The results showed that the ubiquitination level of TGF-βR1 increased after the knockdown of SDC-2, and the ubiquitination level of TGF-βR1 decreased after the overexpression of SDC-2 (Figure 6G-H). Taken together, we can conclude that SDC-2 can inhibit TGF-β1 ubiquitination degradation thereby activating the TGF-β pathway.”

• Along the same lines, ambiguity in experimental setup and the methodology lacks clarity in certain areas. For example, in Results section 3.1, paragraph two, you indicate an intention to study the relationship between EMT and cellular function in vitro, using an experiment based on LPS treatment (presumably involving LPS injection in mice, though this is not explicitly stated). However, it is unclear how this approach directly addresses the research question. Later, a conclusion is drawn that LPS induces EMT, but this does not fully address the specific question of epithelial cell function, leaving the connection insufficiently demonstrated.

Response to comment: Thanks for the comments. We apologize for the misunderstanding caused by the unclear narrative. The second paragraph of Result 3.1 was validated using human-derived Sv-huc-1 at the in vitro level. We plotted the correlation curve using the WB relative protein expression of the EMT indicator as the horizontal coordinate and the fluorescence intensity at which leakage occurs as the vertical coordinate.
• The figures predominantly show a reduction in epithelial marker expression, accompanied by an increase in mesenchymal markers, which suggests EMT induction. However, additional clarification would strengthen this interpretation.
Response to comment: Thanks for the comments. The current confirmation of EMT in non-tumorigenic diseases mainly rests on functional validation, so we first validated the effect of EMT on urination in mice and the effect of EMT on the barrier function of epithelial cells in Figure 1. In the subsequent validation process we also validated the progression of EMT by altered urination in mice (Figure 2D-I), and epithelial cell anti-leakage function (Figure 4I, Figure 5I).
• Much of the data relies on pharmacological inhibition of HPSE via OGT2115, but no complementary genetic knockdown model (e.g., siRNA against HPSE).

Response to comment: Thanks for the comments. We added this part in Figure S1. Since OGT2115 is an HPSE inhibitor, we chose overexpression of HPSE among knockdown and overexpression for validation. The experimental results showed that overexpression of HPSE promoted EMT progression. In the article description is as follows:

“We overexpressed HPSE and validated it using WB (Figure S1A). Overexpression of HPSE promoted EMT progression, epithelial dysfunction, and increased fluorescence leakage occurred (Figure S1B-D). We knocked down SDC-2 on the basis of overexpression of HPSE (Figure S1E).The EMT-promoting effect exerted by overexpression of HPSE was counteracted by SDC-2 knockdown (Figure S1E-F).Epithelial function was restored and fluorescence leakage was reduced after SDC-2 knockdown (Figure S1G).”

• Specific details are missing, such as the number of mice used for each experiment and the control conditions, including necessary controls for siRNA (e.g., Si-scramble control for SDC-2 knockdown). The absence of these details weakens the methodological rigor
Response to comment: Thanks for the comments. Due to our inadvertent misinterpretation, we have revised the content to increase the rigor of the study.
• Additionally, there is a lack of precision in terms of the number of mice used in each experiment. On page 5, Section 2.12, the statistical methods section mentions standard deviations and ANOVA tests but lacks detailed information on sample sizes for each experiment. This makes it challenging to assess the robustness of the findings. Please address these issues to enhance the rigor of your research and provide more details on group sizes, randomization, also blinding methods, particularly regarding pain and urodynamic assessments
Response to comment: Thanks for the comments. We have added the sample size, and the statistical analysis methods have been added to the figure legends.
• Several figures, including 2A-B, lack clarity and are sometimes unreadable, which diminishes the impact and interpretability of the results. High-resolution images should be provided to ensure that all figure details are visible. Furthermore, the contrast and clarity of fluorescence or histological images should be improved to enhance figure readability.

Response to comment: Thanks for the comments. We have re-uploaded the image.
