# Supplementary material for: Inhibition of HPSE/SDC-2 axis-induced epithelial-mesenchymal transition for treating IC/BPS
Source: PLoS One. 2025 May 23;20(5):e0321730. doi: 10.1371/journal.pone.0321730 (PMC12101628; doi:10.1371/journal.pone.0321730)
Supplement: Attachment — Submitted filename: Response_to_Reviewers_auresp_2.docx [file pone.0321730.s004.docx]

Dear Editors and Reviewers:

Thank you for your letter and for the reviewers’ comments concerning our manuscript entitled “Inhibition of HPSE/SDC-2 axis-induced epithelial-mesenchymal transition for treating IC/BPS” (ID: PONE-D-24-45975). Those comments are all valuable and helpful for revising and improving our paper, as well as the important guiding significance to our research. We have studied the comments carefully and have made corrections, which we hope to meet with approval.

The revisions in the manuscript are in red fonts, and responses to the reviewers' comments are in blue fonts.

Reviewer #1: Thank you for considering my previous comments. However, there are still several issues that need to be addressed:

Introduction Section:

• You added some epidemiological data as requested by Reviewer #2, but it would be helpful to provide more specific details, particularly regarding prevalence data (e.g., breakdown by gender, age, and geographical region).

Response to comment: Thanks for the comments. We have added epidemiological data as follows:

“The prevalence of IC/BPS ranges from approximately 2.7% to 6.5%, with women being affected at a rate ten times higher than men. The global prevalence of IC/BPS is approximately 300 per 100,000 women, with the prevalence among men being about 10%-20% of that in women. When considering only the presence of symptoms suggestive of IC/BPS, the incidence rates for both men and women may increase by more than 10 times.”

• To ensure clarity for unfamiliar readers, please specify that the study focuses on HPSE-1 (the enzymatically active form), rather than HPSE-2.

Response to comment: Thanks for the comments. We have specified that our study focused on HPSE-1 as follows:

“There are two forms of heparinase: heparinase-1 (HPSE) and heparinase-2, with heparinase-1 exerting the primary biological functions, which our study focused on.”

Results Section:

• Abbreviations are missing in some areas. It would be beneficial to define them in the results section for easier readability (e.g., CYP mice are described in the Materials and Methods section but could be redefined at the beginning of the paragraph). Additionally, abbreviations in the figures, such as NC, CON, etc., should be included.

Response to comment: Thanks for the comments. We have added this section.

• As per Reviewer #2's request, it would be helpful to include a brief introduction at the beginning of each subsection, outlining the specific objectives of the experiments and the methods used (e.g., "To answer this question, we employed the following methods...").

Response to comment: Thanks for the comments. We have added this section.

Section 3.1:

• The title of this section is misleading because it discusses results from mice, not just in vitro experiments.

Response to comment: Thanks for the comments. We have corrected the following in the original text:

“EMT correlates with urination frequency in mice and barrier function in urothelial cells.”

• It would be helpful to briefly explain the use of LPS for in vitro studies. Although this information is in the Materials and Methods section, it would be clearer if restated in the Results section.

Response to comment: Thanks for the comments. We have added this section as follows:

“We then constructed an urothelial EMT model in vitro by continuously stimulating Sv-huc-1 with LPS for 24 hours to further investigate the relationship between EMT and urothelial cell function.”

• The conclusion in this section is somewhat confusing. While the results pertain to in vitro studies, the conclusion seems to mix in vivo and in vitro findings. A summary of both in vivo and in vitro results would make this clearer before the conclusion.

Response to comment: Thanks for the comments. We have added this section as follows:

“This result demonstrates a correlation between the occurrence of EMT and urothelial cell barrier function, suggesting that the progression of EMT disrupts the epithelial cell barrier function. Taken together, these experimental findings indicate that EMT is associated with urination frequency in mice and barrier function in urothelial cells.”

Section 3.2:

• The conclusion specifies the role of OGT2115 in improving urinary symptoms. It would be beneficial to include the name of the inhibitor in the section title for clarity.

Response to comment: Thanks for the comments. We have added this section.

• It should be clearly stated when analyses were performed on human samples compared to mice. For example, is the GSE57560 dataset from human or mouse samples?

Response to comment: Thanks for the comments. We have added this section as follows:

“The interstitial cystitis dataset GSE57560 was downloaded from the GEO public database, containing 12 interstitial cystitis and 4 control bladder biopsy tissues from humans.”

• For Figures E and F, it would be helpful to add more detailed legends to aid in the interpretation of the data.

Response to comment: Thanks for the comments. We have added this section as follows:

“E: Fixed volumes (5, 10, 20, 50, 100 μl) of mouse urine were dropped onto filter paper for experimental use. ImageJ was used to calculate the area, and an area-volume standard curve was plotted, resulting in the formula: Volume = Area * 12.674. F: Based on the area of mouse urine spots, quantitative analysis was conducted on the frequency of urination, average urine volume, and total urine volume in mice (n=5).”

Section 3.3:

• Figure 3A demonstrates a downregulation of HPSE under OGT2115 treatment, not inhibition. Please use the correct terminology.

Response to comment: Thanks for the comments. We have corrected the following in the original text:

“At the in vivo level by WB assay, OGT2115 was shown to down-regulate the expression of HPSE and SDC-2 (Fig 3A).”

• It may be useful to compare the condition with OGT2115 to control samples to determine whether there is a full rescue of the epithelial phenotype with the treatment (Figures 3B-C and 3E-G).

Response to comment: Thanks for the comments. We have added this section.

Section 3.4:

• OGT2115 is used to inhibit HPSE in vitro. Cytotoxicity assays should be included in this section (or in supplementary data), and the controls need to be clearly specified. Was OGT2115 resuspended in PBS or DMSO? How were the CCK-8 assay results normalized? Is cytotoxicity caused by DMSO or by OGT2115 itself? Furthermore, in the authors' responses to previous requests, their graph did not display the data for 50% cell mortality. Did they extrapolate this data (e.g., 36.26 µM determined as the dose for 50% cell death)? Greater rigor is needed in this experiment.

Response to comment: Thanks for the comments. We did not validate the cytotoxicity of OGT2115 at higher concentrations since the highest concentration of OGT2115 we used was 20 μM. We plotted fitting curves and extrapolated IC_50_ values based on the available data. We have added this section as follows:

“Initially, we used the CCK8 method to measure the cytotoxicity of OGT2115, with the control group receiving DMSO as the solvent. After CCK8 incubation, we measured the OD values, subtracted the blank well value (only culture medium added) from each well, and divided by the control group to obtain the percentage of live cells. After importing the data into Prism, we plotted a fitted curve using the logarithm of concentration as the x-axis and cell survival rate as the y-axis. From this, we calculated an IC_50_ value of 36.26 μM (Figure 4A).”

• In this section, HPSE activity is modulated through pharmacological inhibition, but to strengthen the results, HPSE knockdown experiments should complement these findings (as requested by both Reviewer #1 and Reviewer #2). HPSE knockdown has been proposed as a method to confirm OGT2115's effects and exclude potential off-target effects. However, the HPSE overexpression experiments already conducted are useful and strengthen the results.

Response to comment: We thank the reviewers for their acknowledgement of our experimental protocol. Since an inhibitor of HPSE was used in the experiment, we considered overexpression of HPSE in the subsequent validation to better illustrate the reliability of the conclusions.

Section 3.6:

• Thank you for incorporating our comments to investigate a potential mechanism by exploring the TGF-β pathway.

• The first paragraph could be better integrated into the previous one discussing the role of SDC-2 and its interaction with HPSE for more coherence.

Response to comment: Thanks for the comments. We have added this section as follows:

“The above experiments demonstrate that SDC-2 is a downstream protein that binds to HPSE, but the specific binding site remains unclear. Therefore, based on the HS chain region of SDC-2, we designed truncations (Fig 6A).”

• For readers who may be unfamiliar with the TGF-β pathway, it would be helpful to briefly describe it and explain the rationale behind studying Smad2.

Response to comment: Thanks for the comments. We have added this section as follows:

“Transforming growth factor-β (TGF-β) is a profound factor that promotes fibrosis and EMT, and it is also a downstream signaling pathway of HPSE. When TGF-β1 binds to its receptor, TGFβR1, it can phosphorylate the downstream protein SMAD2. Phosphorylated SMAD2 can bind to SMAD4 and enter the nucleus, activating the signaling pathway through the canonical pathway.”

• The sentence, "This result suggests that the activating effect of SDC-2 on the TGF-β pathway is not through the promotion of TGF-β1 secretion, but may exert its biological function through its receptor," would benefit from further clarification. Please explain the underlying hypothesis more explicitly.

Response to comment: Thanks for the comments. We have added this section as follows:

“The above results show that even with exogenous addition of TGF-β1, the TGFβ signaling pathway cannot be significantly activated after knocking down SDC-2, while the activation effect of TGF-β1 is significantly enhanced after overexpressing SDC-2.”
