# Supplementary material for: Inhibition of HPSE/SDC-2 axis-induced epithelial-mesenchymal transition for treating IC/BPS
Source: PLoS One. 2025 May 23;20(5):e0321730. doi: 10.1371/journal.pone.0321730 (PMC12101628; doi:10.1371/journal.pone.0321730)
Supplement: Attachment — Submitted filename: Response_to_Reviewers_auresp_3.docx [file pone.0321730.s005.docx]

Dear Editors and Reviewers:

Thank you for your letter and for the reviewers’ comments concerning our manuscript entitled “Inhibition of HPSE/SDC-2 axis-induced epithelial-mesenchymal transition for treating IC/BPS” (ID: PONE-D-24-45975). Those comments are all valuable and helpful for revising and improving our paper, as well as the important guiding significance to our research. We have studied the comments carefully and have made corrections, which we hope to meet with approval.

The revisions in the manuscript are in red fonts, and responses to the reviewers' comments are in blue fonts.

Reviewer #1: Thank you for taking my previous comments into consideration. The reading and comprehension are improving, but there are still a few mistakes or misunderstandings that need to be addressed.

- Please be careful: in the introduction section, you mention "heparinase" instead of "heparanase." This needs to be corrected.

Response to comment: Thanks for the comments. We have corrected this error in the introduction section.

- In section 3.1, I would recommend explaining the CYP group further (What does cyclophosphamide do in mice? Which phenotype does it induce?), similar to the explanation already provided for LPS treatment on Sv-huc-1 cells.

Response to comment: Thanks for the comments. We have added the biological functions performed by CYP in the original text, which are described as follows:

“Following intraperitoneal injection, cyclophosphamide (CYP) is metabolized in the kidney into acrolein, a toxic compound to urothelial cells. This process induces urothelial injury and inflammation, ultimately triggering EMT.”

- Be cautious, as some information is repeated within the same paragraph (e.g., "We added LPS stimulation to construct a cellular EMT model" and "We constructed an EMT model of urothelial cells by stimulating them continuously with LPS for 24 hours in the culture medium"). Avoid such repetitions to improve readability.

Response to comment: Thanks for the comments. We have removed the repetitive language.

Reviewer #3: The study has its merits but more efforts need to be made for the presentation of the manuscript. In the abstract it is not clear what CYP-induced mean? What does the abbreviation mean? Cyclophosphamide? Cyclophilin?

Response to comment: Thanks for the comments. We have included the full name of cyclophosphamide (CYP) in the abstract section.

“Both the increased urination frequency observed in mice with acute cystitis induced by cyclophosphamide (CYP) and the disruption of the anti-leakage barrier in urothelial cells induced by LPS are associated with the occurrence of EMT.”

Be careful when writing heparanase, since I saw "heparinase" with "i" many times in the text. This is not the same enzyme. Replace where needed.

Response to comment: Thanks for the comments. We have rectified this mistake, and we thank the reviewer for pointing it out.

An effort should be made to present the results in a more comprehensive manner. For example, look at the first paragraph of the "Results" section: the authors should explain what is the objective/rationale of the experiment, why they do it (what is the expected outcome) and then to explain how (western blot and voiding spots).

Response to comment: Thanks for the comments. We have added an elaboration on the results in the first paragraph of the results section, stated as follows:

“To investigate the correlation between the frequency of urination in mice and epithelial-mesenchymal transition (EMT), we quantified the number of urinations by counting the urine spots on filter paper in the voiding spots experiment.”

“The epithelial permeability assay was employed to evaluate the barrier function of urothelial cells, where the fluorescent intensity of leakage is inversely proportional to the barrier function.”

In the "Results" section, the title of the following paragraph is not clear: “Inhibition of HPSE ameliorates cystogenesis in CYP-induced cystitis mice EMT”. Also, explain why you check E-cadherin, N-cadherin, vimentin…

Response to comment: Thanks for the comments. E-cadherin, N-cadherin, and Vimentin are three EMT-related proteins that are widely used and validated in EMT-related studies. During our literature review, we found that previous studies on IC/BPS and EMT had adopted these three proteins, which were verified to be expressed in urothelial cells (PMID: 34368980, PMID: 34984178). Therefore, we conducted our research using E-cadherin, N-cadherin, and Vimentin.
